# Design and Test of a Spoke-like Piezoelectric Energy Harvester

**DOI:** 10.3390/mi13020232

**Published:** 2022-01-30

**Authors:** Shan Gao, Qiang Cao, Nannan Zhou, Hongrui Ao, Hongyuan Jiang

**Affiliations:** School of Mechatronics Engineering, Harbin Institute of Technology, No. 92 West Dazhi Street, Harbin 150001, China; gaoshan@stu.hit.edu.cn (S.G.); qiangc1@163.com (Q.C.); znn_hit1995@163.com (N.Z.); jhy_hit@hit.edu.cn (H.J.)

**Keywords:** energy harvester, piezoelectric material, magnetic field, vibration, energy harvesting circuit

## Abstract

With the development of industry IoT, microprocessors and sensors are widely used for autonomously transferring information to cyber-physics systems. Massive quantities and huge power consumption of the devices result in a severe increment of the chemical batteries, which is highly associated with problems, including environmental pollution, waste of human/financial resources, difficulty in replacement, etc. Driven by this issue, mechanical energy harvesting technology has been widely studied in the last few years as a great potential solution for battery substitution. Therefore, the piezoelectric generator is characterized as an efficient transformer from ambient vibration into electricity. In this paper, a spoke-like piezoelectric energy harvester is designed and fabricated with detailed introductions on the structure, materials, and fabrication. Focusing on improving the output efficiency and broadening the pulse width, on the one hand, the energy harvesting circuit is optimized by adding voltage monitoring and regulator modules. On the other hand, magnetic mass is adopted to employ the magnetic field of repulsive and upper repulsion–lower attraction mode. The spoke-like piezoelectric energy harvester suggests broadening the frequency domain and increasing the output performance, which is prepared for wireless sensors and portable electronics in remote areas and harsh environments.

## 1. Introduction

The portability of electronic products and wireless sensors has been greatly improved since the development of microelectronics science and nanoscience technology in the new century. The usage of traditional chemical batteries, due to their limited working time and big volume, cannot satisfy the requirement of the power supply for these devices. One alternative way of providing effective electricity is using a micro energy harvester, which includes photothermal energy, vibration energy, static electricity, wind energy, and magnetic field energy converted into electricity, which can be stored in specific equipment. To realize the transformation, a variety of new energy-converting materials [1,2,3,4], structures, and systems [5,6] are utilized based on versatile mechanisms, including the photovoltaic effect [7,8], electrostatic harvesting [9,10], piezoelectric effect [8,11,12,13], electromagnetic induction effect [14,15], magnetostrictive effect [16], etc. It is noted that the piezoelectric effect has been studied for its high power density and easily controllable characteristics in the application [17], such as harvesting machine vibration and kinesthetic movement [18,19].

A cantilever beam [20] is a basic piezoelectric energy harvester (PEH) due to its simple structure and easy manufacturing. The narrow bandwidth can be promoted by array piezoelectric cantilever beams [21,22], segmented cantilever beams [23], magnetic adjustment [24,25,26], multi-directional energy harvesting [27,28], etc. In addition, new flexible composite piezoelectric materials [29,30] have been employed to replace the traditional piezoelectric ceramics due to their frangibility and limitations. In order to obtain more effective power of the micro energy collector and better satisfy the load requirement, a large quantity of research has combined the piezoelectric and electromagnetic mechanisms [31,32] for capturing the vibration and electromagnetic energy, increasing power output, and broadening the frequency bandwidth [33,34]. These methods have developed the application of battery substitution technology in wearable and medical equipment [35,36].

Although the cantilever beam is the simplest structure of a PEH, it has some limitations. For example, its narrow working frequency bandwidth results in lower power density output on some occasions. Additionally, in many cases, a large amplitude leads to material damage and fatigue failure. Therefore, a spoke structure of the energy harvester is proposed to overcome the problems listed above, which has been widely shown in wheel hubs of vehicles. Generally, it has two different types. Huang et al. [37] have demonstrated a kind of spoke structure energy harvester. The vibrator consists of four trapezoid parylene-C beams, which are fixed to a square silicon board with a pass hole in the middle. Its resonant frequency is 615 Hz and the output voltage reaches 368 mV with PVDF piezoelectric material. The output power is calculated as 0.288 μm with the added resistance of 150 kΩ. The other type was studied by Firdaus et al. [38], who introduced a spoke-type permanent magnet generator. The vibrator is a square board with eight trapezoid hollows evenly distributed around the center. The output voltage of this kind of spoke-type energy harvester can reach 13 V. By adding 2 Ω resistance to the circuit, the output power density can be 0.99 W/cm^3^.

In this work, a spoke-like piezoelectric energy harvester (SPEH) is firstly designed and fabricated, with detailed structure, materials, and fabrication. Then, the mathematic model is built and the simulation is analyzed with theoretical output on voltage and power of the fixed beam structure of the SPEH, focusing on the actual nonlinear. Next, experiments are conducted for further discussion and certification compared with the theoretical results. Moreover, an optimized energy harvesting circuit, by adding voltage monitoring and regulating modules, is tested in order to improve output stability and efficiency. In addition, the effect of internal impedance on the closed-circuit output voltage is controlled, which ensures a direct current, stable voltage, and high power density electrical output signal. Furthermore, magnetic mass along with the magnetic field are adopted to achieve broadening the frequency domain for various vibration applications, mainly due to the extra repulsive and attractive mode, and this accordingly results in high power density.

## 2. Materials and Methods

### 2.1. Configuration of the PEH

**Figure 1:**
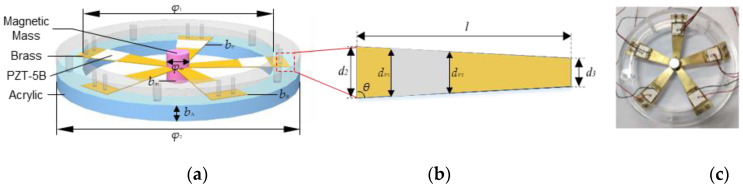
Schematic diagram of the SPEH. (**a**) Simulation schematic diagram of SPEH structure. (**b**) Dimensions of a single trapezoidal brass sheet. (**c**) Experimental prototype of SPEH vibrator.

In this study, a spoke-like structure of a piezoelectric energy harvester is proposed, as shown in Figure 1. A spoke-like vibrator consists of five uniform trapezoidal brass sheets with piezoelectric material of lead zirconate titanate piezoceramic (PZT-5B) layers. These five sheets are fixed symmetrically by two magnets and two toroidal acrylic fastening plates at both ends. PZT-5B and toroidal plates act as the electricity-generating sources and the fixed bases, respectively. The magnets are attached to the sheets as the tip mass to adjust the natural frequency. In addition, they also provide a magnetic field to affect the dynamic characteristics of the system. In conclusion, the SPEH is a kind of “five-end fixed and central mass shared” configuration, which means the vibrator starts to vibrate up and down along with the magnets in the axial direction. The detailed parameters of the SPEH structure are shown in Table 1.

### 2.2. Measurement Methods of the SPEH

**Figure 2:**
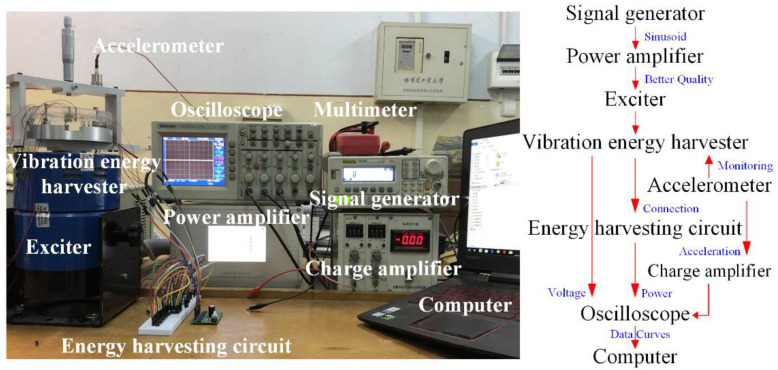
Schematic diagram of experimental test system.

The piezoelectric vibration energy harvesting experimental system is shown in Figure 2. A signal generator provides a sinusoidal excitation to the vibration exciter to achieve the basic vibration. The sinusoidal signal flows through the amplifier and amplifies the signal for easy observation. Then, the exciter drives the harvester to vibrate. The piezoelectric vibrator of the energy harvester deforms when it is subjected to external vibration, which causes the piezoelectric materials to generate electric charges and achieve the purpose of energy collection. An accelerator is attached to the base of the harvester for observing the output acceleration, which is recorded by the charge amplifier and oscilloscope to validate whether the output acceleration matches the output of the signal generator. Therefore, the oscilloscope is used to capture the voltage, power, and acceleration output. The harvester can be directly connected to the oscilloscope with open-circuit voltage output or connected to the oscilloscope through an energy harvesting circuit with closed-circuit power output. All the output signals are displayed on a computer.

## 3. Modeling and Analysis

**Figure 3:**
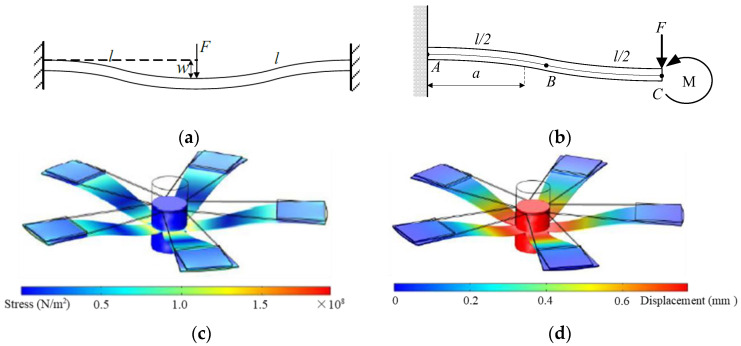
Schematic diagram of the SPEH model and colored pattern. (**a**) Fixed beam simplified model (cross-section of SPEH vibrator). (**b**) Fixed beam simplified model (single trapezoidal beam of SPEH vibrator). (**c**) Colored pattern of stress. (**d**) Colored pattern of displacement under external force.

The simplified model and simulation results of stress and strain of the SPEH are shown in Figure 3. It demonstrates the vibrating condition with a cross-section diagram, which is abstracted as a single beam. According to the colored patterns of Figure 3c,d, the stress and displacement are displayed at the maximum deformation. In addition, it can also demonstrate the movement of the SPEH when it vibrates. It vibrates up and down with a flapping movement with every beam deforming as shown in Figure 3b.

The “five-end fixed and central mass shared” model (shown in Figure 1a) can be simplified to a fixed beam clamped on both ends, approximated to a linear system under the premise of small deformation, as shown in Figure 1b. The deflection *w* at point *C* of the cantilever beam is:

(1) $$w_{C}=w_{F}-w_{M}=\frac{Fl^{3}}{3EI}-\frac{Fl^{3}}{4EI}=\frac{Fl^{3}}{12EI}$$

where *l* is the length of the beam, *E* and *I* are Young’s modulus and moment of inertia, respectively, *F* is the equivalent external force, *F* = *F_Total_*/*N*, in which *F_Total_* is the total external force including the added force and the gravity of the central mass, *N* is the quantity of the fixed beams.

The total stiffness *k_Total_* is derived through a linear superposition of the overall system:

(2) $$k_{Total}=Nk=\frac{F_{Total}}{w_{C}}=\frac{60EI}{l^{3}}$$

Additionally, the equivalent mass of the cantilever beam system, *m_e_*, can be obtained by the energy method:

(3) $$m_{e}=m+\frac{33}{144}m_{b}$$

where *m* and *m_b_* are the mass of the magnet and beam, respectively. Therefore, the natural frequency of the system *ω_n_* can be calculated as:

(4) $$\omega_{n}=\sqrt{\frac{k_{Total}}{m_{e}}}=\sqrt{\frac{60EI}{l^{3}\left(m+\frac{33}{144}m_{b}\right)}}$$

The bending moment, *M_a_*, at the central cross-section with a distance *a* from the toroidal end of the beam is:

(5) $$M_{a}=Fa-\frac{Fl}{2}=\frac{F}{2}\left(2a-l\right)$$

Figure 3b shows that the total moment of the AB section is clockwise when the stress of the beam is in a stretched state, whose large deformation suits the piezoelectric materials generating more charges. Thus, PZT-5B should be attached close to the fixed toroidal end of the beam for its large deformation.

In the case of large deformation, the nonlinearity of the piezoelectric trapezoidal fixed beam system can be described as [37]:

(6) $$F=k_{b}w+k_{s}c^{3}$$

where *w* is the deflection of the system, *k_b_* and *k_s_* are linear and nonlinear stiffness factors, expressed as

$$k_{b}=\left(\frac{\pi^{4}}{6}\right)\left[\frac{E\left(d_{2}+d_{3}\right)h^{3}}{16l^{3}}\right]$$

and

$$k_{s}=\left(\frac{\pi^{4}}{8}\right)\left[\frac{E\left(d_{2}+d_{3}\right)h}{16l^{3}}\right]$$

respectively, where *h* is the thickness of the beam.

The deformation of the beam at the center point subject to external force is defined as *w* = *A*sin*ωt*. Therefore, the differential equation of motion of the system can be expressed as:

(7) $$m_{e}\ddot{w}+c\dot{w}+k_{b}w+k_{s}w^{3}=m_{e}A_{CC}\cos\left(\omega t+\varphi \right)$$

where *ω* is the ambient excitation frequency, *A_CC_* is the amplitude of the applied acceleration load, and *φ* is the phase of vibration.

As the magnets are introduced to add an electromagnet field, the piezoelectric vibration electromechanical coupling equation is [38]:

(8) $$m_{e}\ddot{w}+c\dot{w}+k_{b}w+k_{s}w^{3}+\Theta_{bc}U\left(t\right)=m_{e}A_{CC}\cos\left(\omega t+\varphi \right)$$

where Θ*_bc_* is the backward coupling constant and Θ*_bc_U*(*t*) is the piezoelectric coupling force generated by the voltage *U*(*t*).

## 4. Results and Discussion

Without adding a follow-up circuit (e.g., filter, rectifier, regulator circuits, etc.), the harvester can be seen as an open-circuit mode. On the contrary, the harvester can be seen as a closed-circuit mode with a follow-up circuit. These outputs can be expressed in the form of voltage or power (adding resistances to transfer voltage into power). In this section, the harvester is excited by a sinusoidal vibration with an acceleration of 1 g amplitude in the axial direction of the magnets. Therefore, open- and closed-circuit mode experiments are conducted to derive the output performance.

### 4.1. Open-Circuit Response Experiment

**Figure 4:**
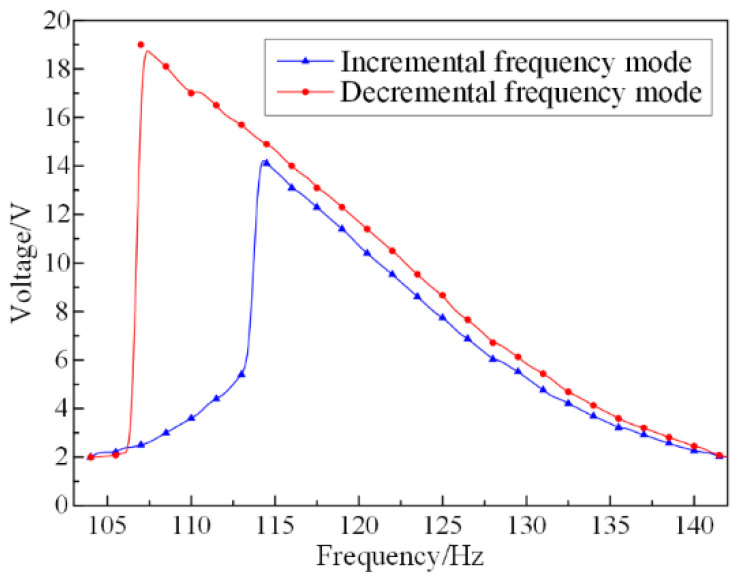
Dependency of the voltage on different frequency mode of the SPEH.

In the open-circuit frequency response experiment, the voltage outputs are initially measured under no-load and no-magnetic field conditions in order to explore the output characteristics of the SPEH. It is conducted with two aspects: one is an incremental mode, which tests the output voltage on frequencies from small to large values; the other is decremental mode, an opposite frequency testing direction from incremental mode. The dependency of output voltage on excitation frequency is shown in Figure 4.

It can be clearly seen that the open-circuit voltages in the two modes have similar jumps and sudden changes, although the jumping points are different. This is due to the existence of axial force in the system in the large deformation vibration, which reflects the characteristics of the nonlinear vibration system. The vibration amplitude response of a nonlinear system (shown as the open-circuit voltage response in Figure 4) is related to the initial vibration conditions. At the same time, for the state with a large vibration amplitude, and for slight disturbances of the external environment, the vibration state may change suddenly, and the amplitude jumps from one state to another. Therefore, if the vibration system is tested with different frequency change modes, under the same external excitation load, the voltage output obtained will also have a significant difference. This assumes that the decremental frequency mode is performed in the system and takes a certain voltage as the effective voltage, for example, 2 V. Therefore, the frequency domain where the voltage exceeds 2 V is regarded as the effective bandwidth. According to the curve, the bandwidth is almost 40 Hz, meaning that the SPEH can maintain a high output level for a large frequency domain. Therefore, the SPEH can respond to a wide range of ambient frequencies with high voltage output. This superior advantage may have wider applications after optimizing the volume and mass of the SPEH.

### 4.2. Closed-Circuit Response Experiment

**Figure 5:**
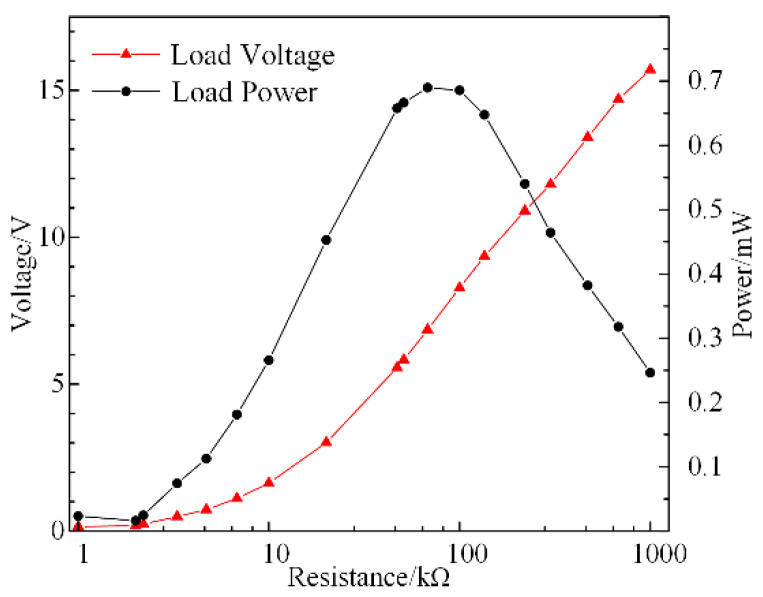
Closed-circuit voltage and power versus resistance of SPEH.

A matching impedance test is required since the internal resistance is relevant in the closed-circuit response experiment. In accordance with Figure 4, the excitation frequency is set at the value of the resonant frequency of 107 Hz. The variation in the closed-circuit voltage with the load resistance is shown in Figure 5.

**Figure 6:**
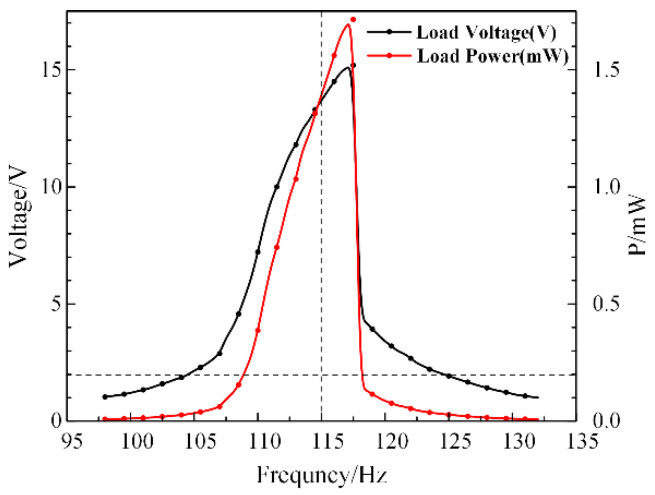
Dependency of the voltage on frequency domain of the SPEH.

In Figure 5, the curve of the voltage is shown as a continuously rising trend. This is because the generated charges of the SPEH under a fixed frequency are certain. Hence, an increase in resistance resulted in an increase in voltage. In principle, the larger the resistance that is added, the higher the output voltage. The curve of the power has a maximum value, suggesting an equivalent internal resistance approximately equal to the outer load. This means that the optimal impedance matching resistance is the load resistance corresponding to the peak power. For example, under certain load conditions, the equivalent matching impedance of the system can be quickly obtained from Figure 5, which is about 80 kΩ. In order to obtain more electrical energy, the stress around the piezoelectric material can be increased by increasing the weight of the center mass. In the experiment, parallel connection is utilized to unite the beams in order to sharply reduce the total resistance and gain high power output. Under the parameters in Table 1, the matching impedance of the system is 134.7 kΩ. The optimal impendence result is employed in the frequency response experiments to calculate the output power, which is shown in Figure 6.

It can be derived in Figure 6 that the output capability has significant gaps at a frequency of 117 Hz. The maximum voltage output is 15 V and the power output reaches 1.7 mW. Moreover, the bandwidth of a load voltage higher than 2 V is up to 21 Hz. Compared with the soft characteristic of the open-circuit output voltage curve (Figure 4), the closed-circuit curves clearly show a hard vibration characteristic. Additionally, the maximum output power of the SPEH reaches 1.7 mW under 1 g acceleration excitation with significantly high piezoelectric coupling damping. Moreover, the power density is calculated to be 1.18 × 10^4^ W/m^3^. Therefore, the structure of the SPEH demonstrates better advantages in energy harvesting technology.

Due to the nonlinear system of the SPEH vibrator, large deformation of the brass and PZT-5B would not occur even under high loads, which ensures the structure and output stability of the piezoelectric energy harvesting system. In the meantime, the stress on piezoelectric materials would not vary since the axial force is decomposed, which restricts the charge generation. In order to generate more power, the tip mass of the SPEH can be increased to derive a better pre-deformation amplitude and lower resonant frequency. This is also an efficient method for widening the frequency domain to meet the requirements of different circumstances.

### 4.3. Design and Test of Energy Harvesting Regulator Circuit

The working voltage of commonly used portable electronic devices usually ranges from 3 V to 5 V. However, the PEH has the characteristics of high voltage and low current output, which cannot be directly supplied to the load. Moreover, the vibration in the environment is uncertain and the derived average power is relatively low. Therefore, a certain rectifying and filtering energy storage circuit [35,39,40,41] is required in order to best utilize the output parameters from the PEH and supply more electricity to the external load.

#### 4.3.1. Design of Voltage Regulator Circuit

**Figure 7:**
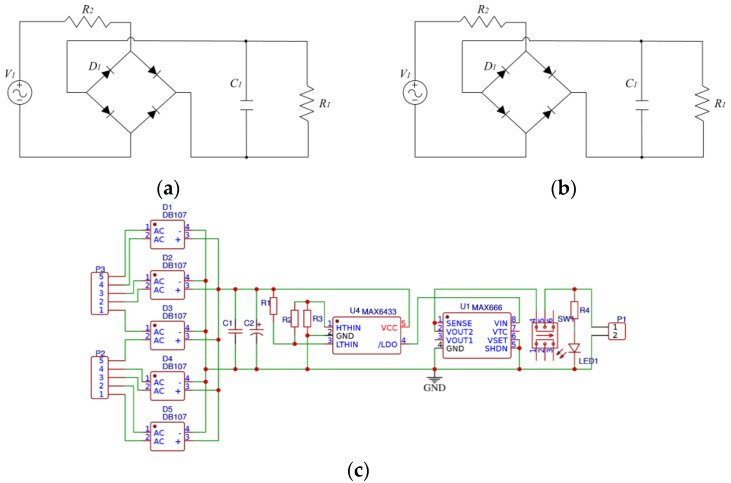
Circuit diagram of the SPEH. (**a**) Traditional energy harvesting circuit. (**b**) Experimental facility. (**c**) Overall rectifier filter energy storage circuit.

The voltage output from the PEH significantly fluctuates under a discontinuous or unsteady vibration source, which is affected by the single-capacitor low-pass filtering effect. Therefore, the traditional energy harvesting circuit shown in Figure 7a cannot meet the requirement of efficient signal processing. Hence, a novel overall rectifier filter energy storage circuit is shown in Figure 7b,c. The electrical signals generated by the SPEH are firstly connected in parallel through the respective rectifier bridges. Then, the signals are low-pass filtered through the ceramic capacitor *C*_1_ and then briefly stored in the electrolytic capacitor *C*_2_. A MAX6433 battery monitor (MAXIM, California, America) is set to achieve real-time voltage monitoring. Moreover, a MAX666 programmable micropower voltage regulator (MAXIM, California, America) is added to the circuit.

The resistances in the circuit shown in Figure 7c are set to *R*_1_ = 1 MΩ, *R*_2_ = 51 kΩ, and *R*_3_ = 100 kΩ. Furthermore, *R*_1_, *R*_2_, and *R*_3_ should be proportionally increased to achieve the purpose of significantly reducing the energy consumption of the rectifier voltage regulator circuit itself for the microenergy collection circuit. The electrolytic capacitor *C*_2_ with a capacity of 1000 uF is the temporary storage container. In order to properly increase the circuit conduction time and equalize the ratio of charge to discharge time, the conduction threshold *V_TRIPHIGH_* (*V_HTH_*) and cut-off threshold *V_TRIPLOW_* (*V_LTH_*) voltages of capacitor *C*_2_ are calculated as:

(9) $$V_{TRIPHIGH}=V_{HTH}=V_{REF}\frac{R_{1}+R_{2}+R_{3}}{R_{3}}=0.615\times 11.51\;V=7.08\;V,$$

(10) $$V_{TRIPLOW}=V_{LTH}=V_{REF}\frac{R_{1}+R_{2}+R_{3}}{R_{2}+R_{3}}=0.615\times 7.62\;V=4.69\;V$$

where *V_REF_* is the internal reference voltage of MAX6433 and *V_REF_* = 615 mV. When *C*_2_ temporarily stores up to 7.08 V and continues for 140 ms, the following MAX6433 chip circuit (MAXIM, Los Angeles, CA, USA) is turned on. The electrical signal is then stabilized at an output of 5 V by the MAX666 chip to the outer loads. With the voltage of *C*_2_ dropping to 4.69 V, MAX6433 is disconnected from the follow-up circuit. Afterward, *C*_2_ continues to begin another procedure of storing energy until the voltage reaches *V*_HTH_ so as to supply energy in cycles.

#### 4.3.2. Output Tests on Energy Harvesting Regulator Circuit

**Figure 8:**
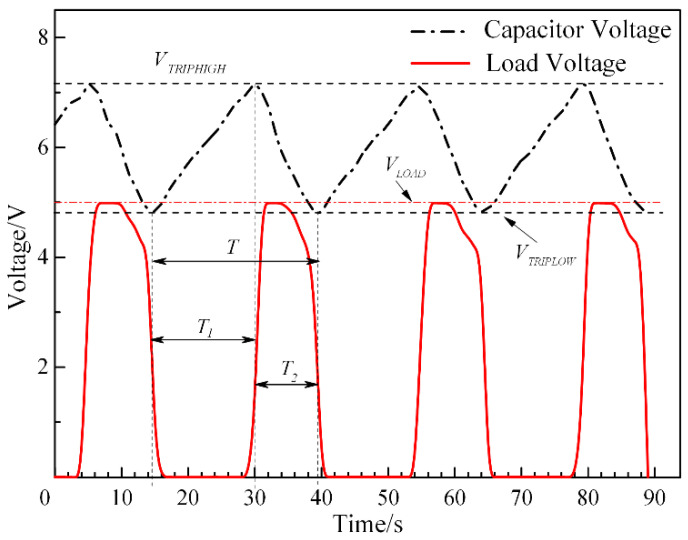
Dependency of the load and *C*_2_ voltage on time domain.

Figure 8 shows that the voltages of *C*_2_ and P_1_ ports vary with the time domain in the energy harvesting regulator circuit experiment. According to the curves, the maximum voltage output is maintained at 5 V of the P_1_ port signal. It can be inferred that the energy collecting circuit proposed in this paper has good rectified and filtered abilities to ensure smooth output performance. The curves show that *V_LTH_* and *V_HTH_* are about 4.8 V and 7.2 V, respectively, which are in good agreement with the calculated values. Since the circuit is turned on with an operating delay of nearly 140 ms, the voltage in the connection state is slightly larger than *V_HTH_*, which is approximately the same as the original designs. In the regulator circuit, the LED indicator is in series with a 10 kΩ load resistor *R*_4_ connected to the P_1_ port as evidence of the power output. It can be seen in Figure 7b that the load circuit turns on and the LED indicator lights up after pressing the switch. When the P_1_ port load circuit is open, the voltage of 4.988 V is consistent with the theoretical voltage of 5 V. In addition, according to the capacitor voltage curve, the charge-discharge cycle *T* of the microenergy collecting and rectifying circuit is 25 s, including 16 s of the capacitor charge storage period *T*_1_ and 9 s of the capacitor discharge charge period *T*_2_.

The voltage curves shown in Figure 8 demonstrate the steady and circulate output tendency of the monitored and regulated energy harvesting circuit. The transient charge storage method is superior in preventing the fluctuation of the variation input signal to the output signal and improving the generated power. For a better performance, a large-capacity capacitor can be selected to store more power for a long period and prolong the discharge procedure. In addition, the values of *V_TRIPHIGH_* and *V_TRIPLOW_* can be adjusted by selecting suitable capacitors and resistances. The energy harvesting regulator circuit has the advantages of not only achieving the purpose of intermittent charging and storing, but also collecting or supplying energy at a high and steady output level from variable voltages for certain durations.

### 4.4. Influence of Magnetic Field on the Output Performance of SPEH

**Figure 9:**
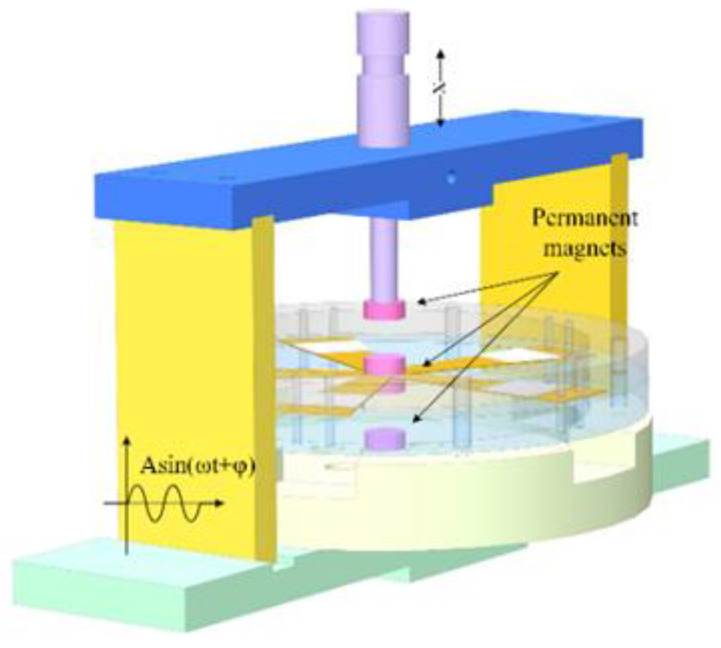
Simplified model of SPEH with external holder for magnetic field experiment.

An external holder is added to the SPEH to explore the influence of the magnetic field on electrical output performance, which is shown in Figure 9. Two sets of cylindrical magnets are coaxially arranged in the axial direction. Taking the upper set of two magnets as an example, the magnetic force *F_magnet_* can be expressed as [42]:

(11) $$F_{magnet}=\left[\frac{B_{r}^{2}A_{c}^{2}{\left(h_{m}+r_{m}\right)}^{2}}{\pi \mu_{0}h_{m}^{2}}\right]\left[\frac{1}{d_{r}^{2}}+\frac{1}{{\left(d_{r}+2h_{m}\right)}^{2}}-\frac{2}{{\left(d_{r}+h_{m}\right)}^{2}}\right]$$

where *d_r_*, *A_c_*, and *μ*_0_ are the distance, common area, and relative permeability of the medium between the two magnets, respectively; *B_r_* is the residual magnetic flux density; *h_m_* and *r_m_* are the height and radius of the magnets, respectively. According to the equation and the experiment condition, *d_r_* is the most convenient and suitable parameter to affect the *F_magnet_*. However, it is necessary to set a safety distance between magnets to prevent damage under vibration, which is set as 10 mm. In addition, cylindrical NdFeB permanent magnets with a diameter of 11 mm and a height of 4 mm are used in this experiment. Additionally, the position and the distance in between are precisely controlled and adjusted by a micrometer.

**Figure 10:**
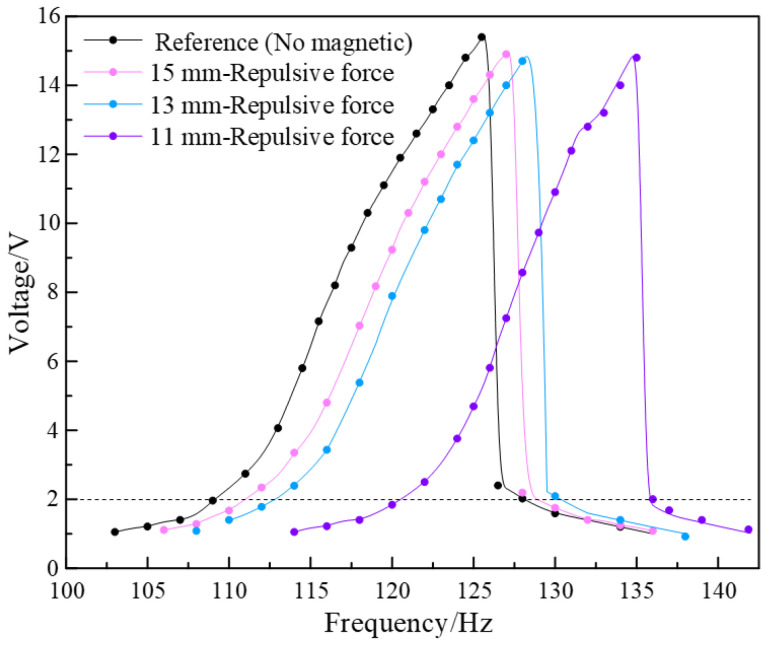
The load voltage in frequency domain under different repulsive modes.

The permanent magnets are distributed symmetrically on both sides of the brass sheet, which act as the tip mass and constituent part of the magnetic field. Extra upper and lower magnets (Figure 9) are added to build the magnetic field during the experiment, with four different combinations of the upper and lower sets of magnets, including both repulsive forces, both attractive forces, upper-repulsive and lower-attractive force, and upper-attractive and lower-repulsive force modes. The imposed magnetic force acts as an extra stiffness of the piezoelectric vibrator in the vibration. Stiffness has an influence on the amplitude of the vibrator, in other words, it affects the resonant frequency domain and output performance. The closer the magnets are, greater rigidity impacted by the magnetic force is attached to the piezoelectric vibrator, and, in consequence, the larger the effective frequency domain is. By adjusting the distance between magnets, the variation in the magnetic force reflects the electromechanical conversion efficiency of the SPEH. In the experiment, the resistance of the closed circuit and the acceleration of the vibration source are set at 134.7 kΩ and 1 g, respectively. Taking upper-repulsive and lower-attractive (UP-LA) forces as examples, the output voltage of the harmonic response experiments is measured. Firstly, the piezoelectric harmonic response of the closed-circuit SPEH under a repulsive magnetic field is tested. The distances between magnets are set as 11, 13, 15 mm, and a reference group without extra magnets is used. The results are shown in Figure 10.

This assumes that a voltage above 2 V is the effective voltage, and the corresponding frequency domains are taken as the effective frequency. It can be calculated from Figure 9 that the output power density of the harvester with repulsive force magnetics is 4.08 × 10^3^ W/m^3^. It can be seen that the relationship between frequency and voltage has the same tendency and maximum value with different frequencies but exhibits a resonant frequency right shift after strengthening of the magnetic force, which shows the nonlinear characteristic. Although the effective frequency domain slightly decreases with the distance shortened, the purpose of broadening the frequency bandwidth can be achieved by changing magnets’ distances from the safety distance to the maximum allowed distance. In a nonlinear system, the frequency domain of the large vibrating amplitude always emerges near the resonant frequency, which is unlike the behavior of the linear vibration system under a specific frequency domain. As a result, the repulsive magnetic field can be added to the SPEH to significantly widen the bandwidth and stabilize the output performance, which demonstrates the same conclusion as the attractive magnetic field.

**Figure 11:**
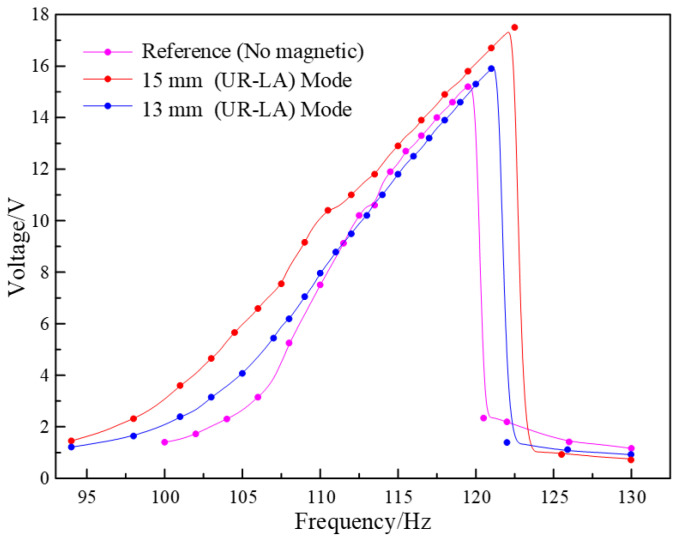
The load voltage in frequency domain under upper-repulsion–lower-attraction modes.

Next, the piezoelectric harmonic response of the closed-circuit SPEH of the different magnetic field orientations is also tested by adding upper-repulsive and lower-attractive (UR-LA) magnetic mode. In this experiment, magnet distances of 13, 15 mm and a reference group are employed as the testing conditions. Additionally, curves of the frequency domain response of the load voltage are shown in Figure 11. It can be calculated that the output power density of the harvester with UR-LA mode magnetics at the highest output voltage is 5.45 × 10^3^ W/m^3^. From the curves, it can be seen that the frequency width and the maximum output voltage are significantly increased. In this way, the amplification of the width and voltage rises to vary degrees as the distance is lengthened further. However, all the magnetic forces are superimposed in the vertical direction under the UR-LA mode. It can be estimated that there is a pre-stress or pre-bend of the vibrator. The piezoelectric vibrator re-establishes an equilibrium position and vibrates under different excitations. In this condition, the amplitude decreases and results in the weakening of the stress variation ranges and output electrical parameters. More seriously, the vibrator almost stops vibrating or is damaged due to the excessive downward magnetic force. As a result, UR-LA mode increases the performance of voltage and frequency width, though the distance between magnets cannot be lengthened significantly.

Among the output results of open and closed circuits with or without the magnetic influence, a comparison can be made to verify the output performance of the spoke-like piezoelectric energy harvester. Additionally, the comparisons of output power densities of published works using PZT piezoelectric materials are also listed. Table 2 shows the power densities of different works.

It can be inferred from Table 2 that the output power densities of the spoke-like piezoelectric energy harvester are at least an order of magnitude larger than the published works, which demonstrates the high ability of charge generation. In addition, the harvester with an external magnetic field shows a higher output, although different modes have different influences, which shows that the magnetic field plays an important role in the vibration condition of the spoke-like harvester. In addition, the microscale device has larger dimensions with the addition of a microprogrammed control unit and power management circuit when leveraged in a wireless sensor network. Therefore, the maximum utilization of the external magnetic field and dimension minification without output performance reductions are the further directions of the research.

The spoke-like piezoelectric vibration energy harvester is designed to leverage the vibration in the ambient environment through the spoke-like mechanical structure to amplify the vibrating amplitude. When the structure vibrates, it causes the piezoelectric layer to experience tension and compression and then the charges are generated. Additionally, the power density can reach 4.08 × 10^3^ W/m^3^. Adding the external magnetic field to enlarge the amplitude of the vibrator can improve the output performance and achieve frequency bandwidth control. The power density under upper-repulsive and lower-attractive modes can reach 5.45 × 10^3^ W/m^3^. This device contributes to producing electricity as an energy harvester for wireless networks and sensors which are used in remote places and places where it is nonreplaceable. Potentially, it can be used as a sensor to perceive force, displacement, or failure in machine operation.

## 5. Conclusions

In this study, a spoke-like piezoelectric vibration energy harvester was designed, fabricated, and tested, and was characteristic of a “five-end fixed and central mass shared” configuration. The proposed SPEH achieves broadband frequency domain and high power output compared with the single cantilever beam and circumferential array cantilever beams. It produces a power of 1.7 mW at 117 Hz under 1 g acceleration, within an effective frequency-domain ranging from 105 to 125 Hz, where the power density reaches 4.08 × 10^3^ W/m^3^. A generalized energy harvesting circuit is optimized by adding voltage monitoring and regulating modules to realize the rectification filter storage and voltage stabilization of the energy recovery circuit. Moreover, the SPEH exhibits a frequency domain of about 30 Hz and maximum voltage output of 17.5 V under an external magnetic field. More than 5.45 × 10^3^ W/m^3^ power density is achieved due to the positive effect of an external magnetic field on output performance, which exceeds at least 8.5% compared to the listed works. The external magnetic field not only can enlarge the output but also can control the frequency bandwidth in required ranges. The SPEH with high output, low resonant frequency, and broadband frequency domain is appropriate for supplying microelectronic and wireless sensors in multiple vibration application scenarios. It also provides a novel idea on broadening the working frequency bandwidth and increasing the efficiency of power output conversion.

## Figures and Tables

**Table 1:** Dimensions of the SPEH components.

Parameter	Value	Unit
Inner diameter of the toroidal plate (*φ*_1_)	100	mm
Outer diameter of the toroidal plate (*φ*_2_)	128	mm
Thickness of the toroidal plate (*b*_A_)	7.5	mm
Length of the trapezoidal sheet’s long side (*d*_2_)	20	mm
Length of the trapezoidal sheet’s short side (*d*_3_)	5.8	mm
Thickness of the trapezoidal sheet (*b*_B_)	0.4	mm
Distance from long side of the trapezoidal sheet to center (*l*)	58	mm
Convergence angle of the trapezoidal sheet (*θ*)	83	°
Length of the piezoelectric layer’s long side (*d*_P1_)	15.0	mm
Length of the piezoelectric layer’s short side (*d*_P2_)	13.9	mm
Thickness of the piezoelectric layer (*b*_P_)	0.4	mm
Diameter of the magnet (*φ*_m_)	11.5	mm
Thickness of the magnet (*b*_m_)	4	mm

**Table 2:** Comparison of output power densities.

Authors	Ref. No	Power Density (W/m^3^)
Gao et al.	Present work	4.08 × 10^3^ (Closed circuit, without magnetic field)
		4.01 × 10^3^ (Repulsive mode, with magnetic field)
		5.45 × 10^3^ (UR-LA mode, with magnetic field)
Xu et al.	[43]	2.80 × 10^3^
Karami and Inman	[44]	5.02 × 10^3^
Jackson et al.	[40]	4.54 × 10^3^
Tian et al.	[41]	1.71 × 10^3^
Lee and Choi	[45]	1.37 × 10^3^

## Data Availability

The data in the paper are in line with MDPI Research Data Policies.
